# Effect of Salt Stress on Growth and Metabolite Profiles of Cape Gooseberry (*Physalis peruviana* L.) along Three Growth Stages

**DOI:** 10.3390/molecules26092756

**Published:** 2021-05-07

**Authors:** Daissy Monroy-Velandia, Ericsson Coy-Barrera

**Affiliations:** Bioorganic Chemistry Laboratory, Facultad de Ciencias Básicas y Aplicadas, Campus Nueva Granada, Universidad Militar Nueva Granada, Cajicá 250247, Colombia

**Keywords:** cape gooseberry, salt stress, growth parameters, metabolic profiling

## Abstract

Colombia is the main producer of cape gooseberry (*Physalis peruviana* L.), a plant known for its various consumption practices and medicinal properties. This plant is generally grown in eroded soils and is considered moderately tolerant to unfavorable conditions, such as nutrient-poor soils or high salt concentrations. Most studies conducted on this plant focus on fruit production and composition because it is the target product, but a small number of studies have been conducted to describe the effect of abiotic stress, e.g., salt stress, on growth and biochemical responses. In order to better understand the mechanism of inherent tolerance of this plant facing salt stress, the present study was conducted to determine the metabolic and growth differences of *P. peruviana* plants at three different BBCH-based growth substages, varying salt conditions. Hence, plants were independently treated with two NaCl solutions, and growth parameters and LC-ESI-MS-derived semi-quantitative levels of metabolites were then measured and compared between salt treatments per growth substage. A 90 mM NaCl treatment caused the greatest effect on plants, provoking low growth and particular metabolite variations. The treatment discrimination-driving feature classification suggested that glycosylated flavonols increased under 30 mM NaCl at 209 substages, withanolides decreased under 90 mM NaCl at 603 and 703 substages, and up-regulation of a free flavonol at all selected stages can be considered a salt stress response. Findings locate such response into a metabolic context and afford some insights into the plant response associated with antioxidant compound up-regulation.

## 1. Introduction

In their natural growth conditions, plants suffer from several biotic and abiotic stresses activating various responses to withstand adverse conditions. One of the more troublesome types of abiotic pressure is salt stress since it affects different growth stages, delaying germination and reducing growth rates such as leaf area, length, and biomass of plants [1]. Furthermore, it interferes with the physiology, the metabolome/proteome, and causes ionic and osmotic stress, which leads to nutrient imbalance, retention of toxic substances, reduction of photosynthetic activity, and formation of reactive oxygen species (ROS) that can produce metabolic dysfunction and even affect genetic material [2]. Such effects can appear separately or jointly, which highly hinders their study.

An evident effect of high salinity is the reduction of biomass and growth of secondary roots and, consequently, the transport of hormones and growth-promoting substances to the leaves would be poor [3]. Leaf primordia and young leaves are highly sensitive to salinity due to their high rates of transpiration and cell division since the tissue is expanding; this leads to a lower number of developed leaves, and leaf abscission and necrosis [4]. Due to high salinity (≥100 mM), the leaf tissue accumulates Na^+^ until toxic levels, leading to the leaf loss and, therefore, photosynthetic area reduction, affecting growth [3]. Additionally, ROS are overproduced in peroxisomes and chloroplasts, which favors oxidative damage in the leaves and interferes with CO_2_ fixation, and increases photorespiration [5].

Abscisic acid (ABA) accumulation, protein kinases of the SnRK type (Sucrose nonfermenting-Related Kinase) activation, and Ca^2+^ loss are some of the acclimating responses to tolerate this kind of stress [6]. This adaptation is the result of the SOS (Salt-Overly-Sensitive) pathway, comprising a series of proteins with transmembrane domains that detect Na^+^ and Ca^2+^ and triggering a regulation mechanism for ionic homeostasis through the salt compartmentalization, mainly in vacuoles, involving its subsequent elimination [7]. Another way of plant response to salt stress is the production of secondary (now currently known as specialized) metabolites. As previously assumed, these compounds did not appear to play a crucial role in the life processes of plants. However, recent studies described and elucidated some of their functions on plant growth and development, even whether they are produced in low quantity (ca. 1% dry weight) [8]. In high-salt events, several secondary metabolites have a protective function against such adverse environmental conditions [9], having roles as osmoregulators, osmoprotectants, and free radical scavengers [10,11]. In this context, the production of free or conjugated phenolic compounds has been highly studied to better understand the strategies and mechanisms to tolerate salt stress [12], since its antioxidant capacity and other benefits are well-recognized (e.g., attractants, UV screens, signaling, structural polymers and defense) [13,14].

A plant belonging to the family Solanaceae with high potential for its cultivation in salinized soils is cape gooseberry (*Physalis peruviana* L.), also known as goldenberry [4,15]. This plant is well-known for the production of sweet fruits, being one of the fruits leading important export rates of fresh matter in several tropical countries [16]. In fact, the cape gooseberry became the second fresh fruit exported after bananas [17]. Cape gooseberry is considered an exotic tropical fruit, being preferred for its flavor, appearance and nutritional quality (e.g., good contents of vitamin A, C, phosphorus, and fiber), and other health benefits related to the presence of some phytochemicals (e.g., phytosterols, polyphenols, fisalins, and withanolides) [18,19]. Indeed, some studies described the hypocholesterolemic, antioxidant, anti-inflammatory, anticancer, and antimicrobial properties of goldenberry fruits, among others [20,21]. *P. peruviana* fruits are mainly used to consume directly or prepare juices or jams [22,23], but aqueous extracts of leaves have been also traditionally used as a diuretic to treat throat conditions and asthma-related problems [24]. Other biological properties of cape gooseberry leaves have also been described, such as antibacterial, cytotoxic, antioxidant, antidiabetic, and antihepatotoxic activities [25,26,27].

Many *P. peruviana* genotypes have been adapted to the edaphoclimatic conditions of specific producing regions (i.e., ecotypes), but a small number of varieties are currently known for the cape gooseberry cultivation. There is the Colombian ecotype, which is characterized by its calyces shape, small fruits (ca. 5 g), bright yellow coloration, and high sugar content [23,28]. Its market acceptability is excellent due to its flavor and appearance, being Colombia, the main exporter of this fruit [29]. Actually, the Colombian Corporation for Agricultural Research (Agrosavia) developed two varieties, namely Corpoica-Dorada and Corpoica-Andina, obtained after in vitro anthers culture. Such varieties are considered the first certified cape gooseberry colombian varieties.

In general, a wide range of agroecological conditions are suitable for several ecotypes/varieties of *P. peruviana*, but the Colombian ecotype grows adequately at 1800–2800 m above sea level, 13–18 °C average temperature, and a well-distributed rainfall between 1000 and 2000 mm per year [30]. In addition, this ecotype requires particular soil conditions to be adapted easily, such as 70%–80% average relative humidity, well-drained soils with a pH between 5.5 and 7.0 and high levels of organic matter [30]. Cape gooseberry crops in Colombia are usually found in salinized soils (electrical conductivity > 4 dS/m) [31]. Actually, one of the main problems in Colombia regarding soil degradation is salinity, which affects its structure, water transport capacity, availability of nutrients for plants and their tissues themselves [31,32].

There are several studies that have investigated the metabolite variations of *P. peruviana* via LC-MS methods [32,33,34], and to the best of our knowledge, only one NMR-based study [16]. However, there is no previous study that has focused on the study of the metabolic response to salt stress along different substages of cape gooseberry plants, which constitutes the novelty of the present study. Thus, the aim was oriented to determine the effect of salt stress on growth and metabolite profiles of *P. peruviana* along three different growth substages. Hence, two different salt treatments, namely low and high salinity (i.e., 30 and 90 mM NaCl, respectively), were applied to *P. peruviana* seedlings to follow phenotypic and metabolic variations during plant development. The plausible relationship between salinity and production of specialized metabolites and the implication of salt stress on growth parameters was then explored.

## 2. Results

### 2.1. Selection of Pre-Germination Treatment

**Figure 1:**
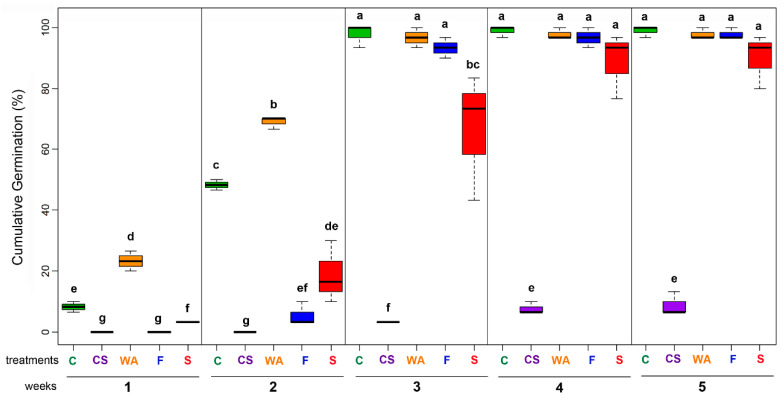
Cumulative germination percentage during five weeks of *Physalis peruviana* seeds subjected to different pre-germination treatments. C: Control (seeds without treatment, green boxes); CS: Commercial (seeds purchased from a local seller, purple boxes); WA: Wood ash (seeds after seven-day storage with wood ash, orange boxes); F: Fermentation (seeds removed after seven-day storage with pulp fruit, blue boxes); S: NaCl (a 60 mM NaCl solution supplied to the seeds three times per week, red boxes). Data expressed as median and interquartile range (n = 3). Different letters over each box indicate significant differences for each %CG according to the post-hoc Tukey test (*p* < 0.05).

In order to homogenize the seedling production *of P. peruviana*, the seed germination behavior was initially examined, using different pre-germination treatments. Cumulative germination percentage (%CG) (radicle > 1 mm) per week was then determined. Figure 1 shows the variations and evolution of %CG over five weeks for each pre-germination treatment. Commercial seeds only reached 8.8% CG, showing an evident viability loss, possibly due to time and storage conditions, which justifies the use of fresh seeds. On the contrary, wood ash promoted the highest %CG from the first week (i.e., 23.3%) and evolved promptly and suitably over time. Thus, its value increased up to 68.9 % at the second week, followed by the seeds without treatment as control groups (48.3%). However, the control group and wood ash reached similar % CG (96.7 and 97.8%, respectively), followed by fermentation and salinized (60 mM NaCl) treatments (93.3% and 66.7%, respectively). These three treatments and control group reached their maximum %CG values until fourth week (> 88%), exhibiting no significant differences (*p* > 0.05) between them according to the post hoc Tukey’s honest significance difference (HSD) test (Figure 1).

The highest standard deviation was exhibited by salinized treatment, whose imbibition and emergence were not uniformly produced. However, salinized treatment reached %CG > 80%, seeds under this condition germinated later than those seeds treated previously with wood ash. After three weeks, control and wood ash and fermentation treatments progressed similarly. However, wood ash promoted a more efficient evolution in germination during the first two weeks. Such profile indicated a germination-promoting effect by wood ash treatment and an early inhibiting effect by salinized and fermentation treatments.

### 2.2. Effects of Salt Stress on Growth of P. peruviana

Owing to the germination-promoting effect obtained by wood ash as pre-germination treatment, leading to fast and homogeneous production of *P. peruviana* seedlings, the study continued through the propagation of *P. peruviana* plants under greenhouse conditions. Seedlings were then treated with two NaCl concentrations (30 and 90 mM, namely low salinity (LS) and high salinity (HS) treatments, respectively), including a control group (without NaCl treatment). Thus, the effect of salt stress on *P. peruviana* growth was initially studied, measuring some growth parameters, such as length (aerial part and roots), leaf area, biomass (aerial part and root), root/aerial part ratio, leaf area/biomass ratio and root ratio, for three selected BBCH-based growth substages of *P. peruviana* [35], comprising a vegetative (209) and two reproductive (603 and 703) substages. These measurements are presented in Table 1. There were no time differences between control and LS groups to reach BBCH substages, but the growth time between control and HS groups was found to be different. Thus, the control group required 90 ± 2 days after transplanting to reach the 209 substage, whereas the HS group needed 15 ± 8 days more to reach this substage. Similarly, 120 ± 4 days after transplanting (i.e., 30 days 209 substage) were required by the control group to reach the flowering stage (603), and there was a time difference (i.e., 18 ± 7 days) between control and HS groups. Finally, slight time differences were found between HS and control groups to reach the 703 substage (195 ± 8 versus 181 ± 5 days, respectively).

Aerial part length showed differences in the reproductive stages. Thus, plants under HS were statistically different (*p* < 0.05) from that of control and LS groups at the flowering stage (603), but such differences were more evident at the fruiting stage (703), specifically between HS and the control group. Differences in root length were also observed between the HS and control groups at the flowering stage, but no significant differences were found at the vegetative (209) or fruiting stages.

The leaf area of the HS group at the flowering stage was also affected by salt treatments, showing a reduction in the mean area and being significantly different (*p* < 0.05) to that of control and LS groups, whereas no significant differences were observed at substages 209 or 703. Additionally, dry-weight biomass of both plant parts (aerial part and roots) presented significant differences between HS and control groups at both flowering and fruiting stages, but no significant differences were found at the 209 substage. The HS treatment had a biomass reducing effect, mostly evident in the root biomass (Table 1).

Some growth parameters-derived indices were then calculated to better appreciate the effect of salt treatments on roots and aerial part (Table 2). Thus, the mean root/aerial (R/A) ratio decreased markedly under salinized conditions at both reproductive stages (more evident at the 703 substage), whereas the mean leave area/total biomass (LA/TB) ratio increased depending on the salt treatment, also more evident at the 703 substage, indicating a balance between tissue development and performance. Similar behavior was observed for the aerial and root mass fractions (AMF and RMF, respectively). However, no clear trend was observed at vegetative and flowering stages, but HS treatment showed positive and negative effects on these indices related to the biomass of the aerial part and roots, respectively, at the fruiting stage (Table 2).

### 2.3. Effects of Salt Stress on Metabolic Profiles of P. peruviana Aerial Parts

The effects of salt stress on metabolic profiles of *P. peruviana* plants along the selected growth stages were studied after ethanolic extraction of the respective aerial parts and subsequent analysis by LC-ESI-MS to obtain the metabolic profiles. The aerial part was selected because leaves contain the highest content of metabolites in *P. peruviana*, especially phenolic-like compounds [17].

**Figure 2:**
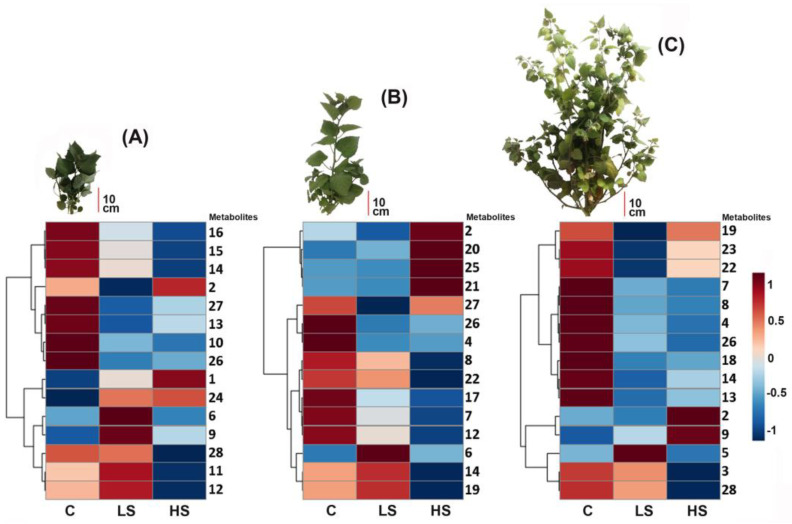
Distribution of the relative abundance of the most contrasting fifteen metabolites detected in extracts of *P. peruviana* aerial parts under salinity conditions, according to the sPLS-DA-derived variable influence on projection (VIP) scores (VIP > 1). This distribution is divided into three heat maps obtained from those profiles of plants at substages (**A**) 209 (vegetative), (**B**) 603 (flowering), and (**C**) 7003 (fruiting). Each heat map is organized by columns for each treatment: HS: high salinity (90 mM NaCl); LS: low salinity (30 mM NaCl); C: control (no NaCl treatment). Each color cell was associated with a normalized (scaled to unit variance, prior heatmap generation) relative abundance of each metabolite, located in the right side of each heatmap, depending on the color scale (dark red: high abundance; dark blue: low abundance). The most contrasting metabolites per growth substage are organized according to the Ward clustering algorithm measuring Euclidean distance, and numbered according to the annotated metabolite list presented in Table A2.

After pre-treatment of raw LC-MS-derived profiles, the peak area of each feature (i.e., metabolite at a retention time) versus observations (i.e., treated plants per substage (*n* = 9) and their replicates (*n* = 12)) were compiled. The resulting metabolic data matrix (feature × observations = 1213 × 108) was initially filtered by the variable influence on projection (VIP) scores after partial least squares discriminant analysis (PLS-DA) over the entire normalized data set. This first classification was performed to explore the distribution of the relative abundance of detected features and select the relevant information from metabolic profiles by the examination of the most contrasting patterns. Hence, the differential comparison of the VIP scores of each feature between salinized conditions led to gather the fifteen most contrasting features per growth substage, based on VIP > 1. Some of these contrasting features were common among growth substages, but other features were exclusively selected for a particular substage. Therefore, for the three test substages, a set of twenty-eight features were statistically selected (fifteen per substage). Such features (**1**–**28**) were annotated at level 3 using their spectral data, which are listed in Table A2. The resulting contrasting patterns were intuitively visualized through heat maps, whose each color cell is associated with a relative abundance of each metabolite to compare salt treatments per substage (Figure 2). These heat maps showed that salt treatment impacted metabolic profiles since important differences (i.e., downregulated and upregulated metabolites) between control and salinized groups were observed. This trend was clearly evidenced by the clustering analysis among these most contrasting features, since two main clusters were observed for all growth substages, comprising downregulated metabolites (i.e., most-abundant metabolites in the control group) as the first cluster and upregulated metabolites by the effect of HS and LC treatments as the second one (Figure 2).

The highest number within the most contrasting metabolites were found to be down-regulated. This fact indicated a depletion of the abundance of some metabolites by the effect of salinized treatments. However, a particular up-regulation of other metabolites differently presented in LS and HS groups along the three growth substages, were also observed. In this regard, LS and HS groups at the 209 substage exhibited four (**6**, **9**, **11**, and **12**) and three (**1**, **2**, and **24**) upregulated features, respectively (Figure 2A), whereas the 603 substage showed three (**6**, **14**, and **19**) and four (**2**, **20**, **25**, and **21**) upregulated features for LS and HS groups, respectively (Figure 2B). Finally, LS and HS groups at the 703 substage resulted in the lowest number of upregulated metabolites, involving one (**5**) and two (**2** and **9**), respectively (Figure 2C).

**Figure 3:**
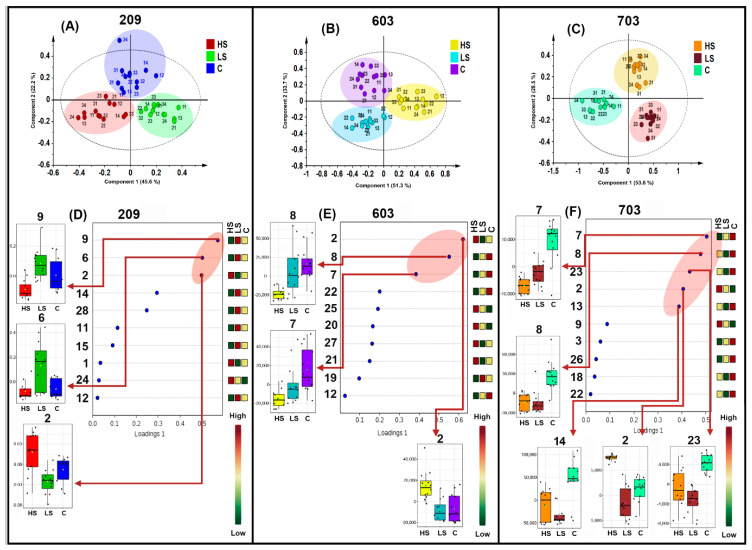
Comparative analysis of the LC-MS data of aerial part-derived extracts from those *P. peruviana* plants collected at different growth substages (209, 603, and 703) under different salt treatments (HS: high salinity (90 mM NaCl); LS: low salinity (30 mM NaCl); C: Control (without NaCl treatment)) by means of sparse partial least squares discriminant analysis (sPLS-DA) to establish the metabolic differences (R^2^ > 0.85; Q^2^ > 0.78). Scores plot supervised by salt treatment: HS: red; LS: green; C: blue for the dataset from plants at the substages (**A**) 209, (**B**) 603, and (**C**) 703. Loadings plot ranking the most-influencing metabolites to discriminate treatments for the dataset from plants at the substages (**D**) 209, (**E**) 603, and (**F**) 703. Metabolites are numbered according to the annotated metabolite list presented in Table A2. The highly top-ranked metabolites per growth substage are highlighted in red ellipses.

Subsequently, in order to facilitate the recognition of those patterns associated with statistical discrimination of salt treatments due to the influence of the differential abundance of particular metabolites, a sparse partial least squares discriminant analysis (sPLS-DA) was then performed, dividing the whole LC-MS-derived dataset into three groups according to the growth stage. The suiting predictive performance of sPLS-DA (i.e., classification error rates < 0.4, cross-validating area under curve > 0.95) for the accurate classification and variable selection of multiclass problems in a one-step procedure has been previously demonstrated, showing more efficiency for feature selection than that of other supervised classification methods [36]. Therefore, sPLS-DA was chosen as the projection-based method to classify and select those most discriminant features under a three-class (i.e., two salt treatment and control groups) comparison scheme. The resulting sPLS-DA-derived score plot for the whole metabolite dataset of those plants at the 209 substage (Figure 3A) showed graphically that categorical variables (i.e., groups framed according to salt treatments) can be discriminated due to differentiated metabolic profiles between them through a well-fitted model (R^2^ = 0.919; Q^2^ = 0.856). Identical pattern for the dataset from plants at the 603 (R^2^ = 0.869; Q^2^ = 0.782) and 703 (R^2^ = 0.951; Q^2^ = 0.861) substages, but a lower dispersion (i.e., a more marked effect) in the score plot related to plants at the 703 substage was observed (Figure 3B,C).

The sPLS-DA-derived loadings plots (Figure 3D–F) were useful to delineate such feature-based differences and select important metabolites, showing the top-ranked metabolites by the discriminating influence through the load vector (loadings) according to the color scale (red: high influence; green: low influence). Thus, ten metabolites were top-ranked for the dataset along growth substages according to their loadings value (>0.3). These metabolite rankings were also related to the quartile-based distribution of metabolite abundances along replicates per salt treatment through respective box plots (Figure 3D–F). The statistical differences of the mean relative abundance of each respective metabolite between salt treatments and control, transformed to the sPLS-DA-derived scores and associated with the median variations, were clearly appreciated, since they exhibited an unusual relative abundance that statistically stands out in comparison to other treatments. Such top-ranked metabolites were related to flavonols and withanolides (free and glycosylated), being responsible for the discrimination of salinity treatments. At the 209 substage, two of the top-ranked metabolites discriminated the LS group (identified as quercetin 3-glucosylgalactoside (**9**) and quercetin 3-robinobioside-7-glucoside (**6**)), whereas one metabolite influenced the discrimination of the HS group (identified as quercetin (**2**)) (Figure 3D). Conversely, the three most-influencing metabolites appeared to discriminate salt treatments at the 603 substage. Two of them were related to withanolides, i.e., withaphysanolide (**8**) and physanolide A (**7**), and differentiated the control group. In contrast, compound (**2**) (already top-ranked in the previous substage) was the highly top-ranked metabolite and its relative abundance was also found to be the highest for the HS group (Figure 3E). At the fruiting stage (703), the respective loading plot (Figure 3F) also had a red ellipse including five top-ranked metabolites that participated in the treatment differentiation for this substage; four of them were related to withanolides (i.e., **7**, **8**, physalin B (**14**), and physagulin D (**23**)) and were more associated to the control group. The five one was compound **2** that again influenced the discrimination of HS group.

## 3. Discussion

Excessive salinity in soils is considered a serious environmental problem that affects the growth and development of plants, from their biochemistry, physiology, and morphology, impacting the production of those commercially important plants [37]. In the first instance, salt stress alters the overall water balance of the plant and, in turn, at the cellular level, disturbs the membranes and proteins, causing metabolic dysfunction [38]. Although salinity could affect different growth stages by characteristic events and conditions, germination is usually a tolerant phase for salinity in crop plants, whose tolerance is manifested by high survival percentage values [1]. However, the germination rate and percentage can be altered by stressful levels of salinity and these responses may vary between species and even cultivars [39]. In the case of the cape gooseberry Colombian ecotype, the present study showed a reduction of the cumulative germination percentage and a germination delay for those seeds that received the salinity treatment (60 mM), compared to the other treatments. After four weeks, the maximum percentage was 88.8%, slightly higher than that reported in studies carried out in Turkey and Brazil, whose germination percentage was ca. 70%, at the same time and salt concentration. Although salinity produced an early germination delay, a cumulative percentage was later comparable to that of the control. Therefore, *P. peruviana* can be considered a salt-tolerant species during this initial stage [15,40]. However, results may diverge depending on the environmental conditions, viability and genetic factors of the test ecotype. Additionally, it is necessary to study each growth stage of the plant against salt conditions to determine if this tolerance is maintained throughout the cycle. Salt stress in glycophytic plants, such as cape gooseberry, is tolerated due to the ability to eliminate the excess of monovalent cations from leaves, since the ion exchange that occurs, favoring the Na^+^ expulsion as an osmotic regulation strategy [41].

The pre-germination treatment based on fermentation has been used as a method of controlling infectious processes in nightshades such as tomatoes. Similarly, in passionflower, it is used to break seed dormancy and promote seedling growth and development [42,43]. However, fermentation effect may fluctuate by the exposure time and the test species since it can be risky by reducing the seed viability [44]. During the first two weeks, the seeds treated with wood ash germinated faster, reaching 68% CG compared to fermentation (5%). In this regard, a higher germination rate could be induced by the action of karrikin-type butenolides possibly present in the wood ash. This family of compounds is common in smoke and in charred plant materials, which act as germination stimulators of some seeds by binding to the KAI2 receptor, as previously studied for other nightshades [45].

Salt stress tolerance is also manifested by growth indicators in the vegetative and reproductive stages. High salinity levels often affect shoot growth more than root growth, so leaf-related parameters may show an evident reduction [1]. Therefore, detailed scrutiny throughout plant growth stages is highly required, since each stage would be a particular physiological and biochemical scenario to respond to salt stress. For instance, it has been reported that the growth of one-month-old cape gooseberry seedlings may be favored by salt concentrations (ca. 30 mM) [31], even concentrations close to 25 mM NaCl do not alter the growth parameters of *P. peruviana* during in vitro cultures [46]. In addition, no growth alterations on roots and stems of 12-week plants were observed using 60 mM NaCl [4], and 90 mM NaCl (9.6 ds/m) can be considered as the optimum salt concentration threshold for stress on the growth of *P. peruviana* [31]. In the present study, the vegetative stage (209) did not show an effect associated with a response to salinity conditions for any of the estimated growth parameters. It is possible that Ca^2+^ supplementation during fertilization helped to alleviate the inhibitory effect of salt on growth at this stage, as reported in other species of the genus *Physalis* and cotton plants [47,48]. However, in the case of the higher stages, such supplementation appeared to be insufficient due to the salt accumulation in the substrate.

In most cases, salinity reduces plant growth because it affects various metabolism aspects. The root part is the first plant organ that faces the imbalance of the ion reserves in substrate. The size reduction in plants at mature stages could be related to cumulative effects of osmotic stress and ionic toxicity due to salinity, manifesting an exacerbated effect as salt concentration increases, as reported for several *Physalis* plants [49]. In general, plants expend energy to counteract stress and try to maintain balanced itself, so growth is postponed. One of the stress-related symptoms is the undersized stems and leaves, which translates into a compensation of photosynthetic activity to capture ROS and other salt-stress-induced metabolites [41]. The effect of salt stress on growth of *Physalis*, using high salt concentrations (>60 mM NaCl), was investigated in previous studies, and an inverse effect between salt concentration and plant growth was found [50]. In other words, higher salt concentrations promoted a lower plant growth since the dry and fresh weight of shoots and plant length were found to be reduced [48]. In this study, a significant reduction in aerial and root length was observed in the high salinity treatment (i.e., 90 mM) compared to the control. Additionally, a reduction in the dry mass of the aerial and root parts, and the other growth parameters in reproductive stages, such as flowering and fruiting, was observed. The results obtained here for the cape gooseberry growth are very important, since the effect of salinized treatments on growth was particularly evident at the commercially important production stages. This fact can be reasoned because, during flowering and fruiting, the plant requires inputs for flower production and fruit filling, so the reduction in the leaf area and leaf biomass directly affects photosynthesis and, in turn, the ability to produce high quality fruits in large volumes [51]. Colombian ecotype of *P. peruviana* was used in the present study and, being a perennial plant, the salt elimination is very important to avoid foliar abscission for ensuring leaves throughout plant life [52].

Salinity induces the up-regulation of some compounds in leaves and roots, such as abscisic acid, which is related to ROS production [53,54], promoting the additional synthesis and accumulation of antioxidant compounds like anthocyanins and other phenolic compounds [55]. Therefore, phenolic compounds, more exactly flavonols (free or conjugated) play an important role during acclimation and/or adaptation of plants to the stressful environment, as they provide unusual qualities for overcoming abiotic stress, such as salinity. In this way, a series of complex metabolic processes are triggered and involved different biological activities such as reducing agents, donors of hydrogen atoms and/or electrons and free radical scavengers [56,57]. The abundance and/or diversity of phenolic compounds varies according to the plant growth stage, being prominent when the plant reached a certain maturity level [19] since this is a way of responding against biotic and abiotic stresses such as toxic levels of salt [1]. Under a condition without abiotic stress, plants have a metabolic heritage in the reproductive stage. However, during a salinized condition, some metabolites may have been depleted in treatments due to salt stress, and the upregulated compounds can be particularly related to a function against such a condition [8]. As observed in the heat maps of Figure 2, 15 most-contrasting metabolites among salt treatments were selected based on VIP > 1 for each growth substage. Some contrasting metabolites were common among the three (3 compounds) and two substages (3 and 11 compounds, respectively), but other contrasting metabolites were found to be unique for a particular substage (14 compounds). Therefore, an entire set comprising 28 contrasting metabolites for the three substages was then compiled (**1–28**, Table A2). These metabolites were observed to be downregulated and upregulated (17 and 11 metabolites, respectively). The non-treated cape gooseberry plants at substage 703 exhibited a higher number of downregulated compounds, i.e., twelve most contrasting metabolites, comprising a phenol (**3**), a flavonol (**4**), and nine withanolides (**7, 8, 13, 14, 18, 19, 22, 23,** and **28**) (Table A2), in comparison to the other growth stages. 

Metabolic differences were evidenced by the sPLS-DA-derived scores plots for each growth stage, indicating that the cape gooseberry responds metabolically to salinity. This separation was more evident along the growth stages since the relative abundance of metabolites for HS treatment decreased during development, while relative abundances in the control group increased, which is rationalized as the normal plant maturity process. For LS treatment at the 209 substage, quercetin-like glycosylated flavonoids **6** and **9** were statistically significant (Figure 3A). This fact indicated that this mixed biosynthetic pathway is activated [58] in combination with the action of UDP-glycosyltransferases, particularly expressed to respond to abiotic stimuli and promote production of biologically-important metabolites, as previously described for model organisms [59]. On the contrary, the free flavonol quercetin was mainly related to the HS treatment at the same growth stage, possibly by a specific response to improve antioxidant features to cape gooseberry plants and tolerate salinized conditions at the vegetative stage. During reproductive stages, the metabolic and physiological plant responses were found to be different. For substages 603 and 703, no metabolites were related to LS groups, whereas quercetin (**2**) discriminated the HS treatment. Unlike the vegetative stage, four withanolides were most abundant in control plants, two at the 603 substage (**7** and **8**) and four at the 703 substage (**7, 8, 14**, and **23**), suggesting that some metabolites are normally produced by this ecotype, but they were downregulated by the effect of salt conditions. From an untargeted metabolomics-based approach, compound **23** was found to be increased in *P. peruviana* fruits from those plants produced under organic systems, possibly due to defensive reasons [32]. In this sense, a clear effect of salt stress on metabolic profiles is related to a depletion of the abundance of some withanolides by salt treatments. Contrarily, withanolides increased considerably (> 80 mg/g dry weight) under high-temperature stress during thermotolerance experiments with *P. peruviana* [60]. On comparing the metabolite variations with growth parameters, in the HS treatment at the fruiting stage, a reduction of the both abundance of certain metabolites and growth was observed, possibly due to the reduction in leaf growth to impulse biosynthesis. Although the vegetative stage was not strongly affected during its growth, a higher number of metabolites were better statistically ranked by the loadings vector, which mainly influenced the discrimination of salt treatments. 

In the present study, under supervised feature classification and selection through sPLS-DA, quercetin-like flavonols were found to be related to the metabolic response against salt stress, even from the 209 substage, since they influenced the statistical discrimination between salt treatments and control. In fact, the box plot of compound **2** exhibited a lesser dispersed relative abundance, indicating a more consistent response against salt stress at the fruiting stage (Figure 3F). Quercetin (**2**) was recently found to mediate salt tolerance in tomato plants through the enhancement of plant antioxidant defense and glyoxalase systems, favoring plant growth and photosynthetic pigment synthesis [61]. It has been reported that the antioxidant activity in fruit trees increases if the plant is affected by salt stress, for protecting tissues against ROS and oxidative damage [62]. Hence, up-regulation of antioxidant compounds is considered a common response induced by salt stress to maintain cellular function and physiological stability of plants [10]. The induction of compound biosynthesis is also related to the accumulation of solutes in cellular organelles to promote osmoregulation. The imbalance caused by osmotic stress disrupts the functionality of the primary metabolism, such as nutrient transport and evapotranspiration. Therefore, the plant responds with a set of secondary metabolism-derived products and modifies its growth to balance physiological processes and withstand salt stress [63]. In this context, since cape gooseberry is moderately tolerant to salinity, the specialized metabolism would be a response to overpass salt stress.

## 4. Materials and Methods

### 4.1. Plant Material

Seeds of *Physalis peruviana* were obtained by direct extraction of ripe fruits of the Colombian ecotype from a local commercial crop. Seeds were separated from the pulp in a mortar with distilled water (DW). Successive washes were then carried out with DW and the seeds were subsequently dried with absorbent paper and stored in paper bags until used [64].

### 4.2. Germination Assays:

A comparison was made on seed germination using four different pre-germination methods, as reported in the literature, including a control. The seeds were disinfected with a 1% NaClO solution for 1 min. Seeds were then placed in germination chambers. The germination chamber consisted of one 150 × 22 mm^2^ Petri dish with absorbent paper. Thirty freshly-removed *P. peruviana* seeds were placed onto the absorbent paper (Uline, Pleasant Prairie, WI, USA). The seeds were watered three times per week with sterile distilled water (SDW), or according to the corresponding treatment. There were three repetitions for each treatment. The total number of seeds for each treatment was 90. The germination chambers were maintained at room conditions (20 °C, sunlight, 12/12 (day/night) photoperiod) to initiate imbibition and subsequent germination. Seeds were considered as germinated if the radicle was visible (at least 1 mm) [15,65]. The cumulative germination percentage was measured every week for 5 weeks. The pre-germination treatments were selected and organized as follows: (1) Fermentation: Seeds were directly stored with the fruit pulp in a lidded bottle with 50 mL of DW during a week in darkness. After this time, the seeds were washed with DW; (2) Wood ash: Seeds were immersed into a 150 mL mixture of DW with 1.0 g of wood ash (obtained from a eucalyptus tree) during a week in darkness; (3) Salinity (NaCl): A 60 mM NaCl solution in SDW was prepared to be supplied to the seeds three times per week; (4) Commercial: Seeds were purchased from a local seller (Copragro S.A.S, Bogotá, Colombia), previously treated with two antifungals (Thiram and Captan) (Syngenta, Chicago, IL, USA) prior packaging; (5) Control: SDW was used to keep the seeds moist inside the germination chambers.

Once the best treatment for rapid and homogeneous germination of the seeds was selected, a new germination procedure was carried out to obtain seedlings with the two cotyledons fully deployed.

### 4.3. Plant Material and Management

This experiment was carried out under greenhouse conditions (average temperature 14.3 ± 5.2 °C, and 81.8% ± 10.3% relative humidity). The seedlings with deployed cotyledons were transferred to blond peat (Pindstrup Plus Orange) (Pindstrup Mosebrug A/S, Ryomgaard, Denmark) in 72-well seedbeds until they reached the 104 substage of the *P. peruviana* BBCH scale [35], with daily irrigation until the substrate is saturated. They were kept inside a tunnel with 50% polyshade. Once desired substage was reached, the seedlings were individually transferred to 8 L plastic bags with a 2:1 loamy-silty soil:rice husk mixture. The soil was previously solarized for five weeks as a disinfection procedure. In addition, the required volume of water was determined to reach the field capacity of the substrate and ensure adequate irrigation. For this, the cylinder volume equation was used as indicated in Equation (1):

(1) $$V=\pi r^{2}h$$

where *r* corresponds to the radius of the cylinder and *h* is the height.

Using a ThetaKit probe (Δ-T Devices Ltd, Cambridge, UK), the percentage of moisture was estimated in the substrate and, knowing the volume of the bag, it was possible to calculate the specific volume of irrigation for each bag, as in Equation (2):

(2) $$V=\frac{\theta_{FC}-\theta_{H\%}}{100}\times V_{bag}$$

where *θ_FC_* corresponds to the expected field capacity, *θ_H%_* is the percentage value of humidity that the probe throws and *V_bag_* is the volume of the bag previously calculated.

Based on soil analysis, three applications of Mainstay Ca 21.4% (Cosmocel Iberica, Zaragoza, Spain) were made, with an application dose of 20 mL/30 L, throughout the culture cycle. The fertilization was managed in two ways: (1) foliar fertilization: provided once per week from substage 209 until the end of the crop cycle (nutrifoliar: 2 cm^3^/L; carrier: 1 cm^3^/L); (2) fertigation: A Hoagland’s solution (prepared as the mixture presented in Table A1), suitable for the cultivation of gooseberry according to soil analysis, was supplied once per week during vegetative stages, and twice per week during reproductive stages. The irrigation of the crop was manual and depended on the percentage of humidity obtained with the ThetaKit probe and the calculation made with Equation (2). This calculation was made for each plant (as biological replicate) three times a week. Weed control and maintenance pruning were performed once per week. The distance between plants was maintained, and the leaves did not touch each other.

### 4.4. Plant Treatments

Three treatments of 0 (control), 30 (low salinity), and 90 (high salinity) mM NaCl were studied over one year. The salt concentrations were applied to the plants every two days. Each experiment repetition comprised 36 experimental units (each experimental unit constituted a plant in an individual bag), corresponding to plants of the same age and the same transplanting time, to ensure twelve replicates per growth substage. They were arranged in 8 L plastic bags, labelled as treatments or control, to involve an entire set of 108 plants. The developmental substages selected for the study included a vegetative stage (209) and two reproductive stages (603 and 703) on the BBCH scale for *Physalis peruviana* [35]. The plants were harvested upon reaching each substage. Plant parts were separated into aerial and root parts.

### 4.5. Growth Parameters

The growth parameters were taken at each harvesting time. Plants were allowed to grow until they reached the substages 209 (nine visible apical bifurcated shoots), 603 (three open flowers), and 703 (three fruits with typical size and shape), according to the BBCH scale [35]. After removal, roots were washed, and the following growth parameters (such as length, leaf area, and dry-weight biomass) were measured and other indices (such as root/aerial and leaf area/biomass ratios, and leaf mass and root mass fractions) were calculated according to previously reported information [66,67].

### 4.6. Extraction of Plant Material

To prepare the ethanol-soluble extract, freshly harvested plant material (aerial part) was rapidly frozen, lyophilized, dipped in liquid nitrogen and ground into a fine powder using a mortar. Dry, ground plant material was extracted with 96% ethanol under stirring using stainless steel beads for 30 min at room temperature. The resulting mixtures were filtered and concentrated under reduced pressure. The resulting raw extracts were stored (maximum 3 days) at −20 °C until analysis.

### 4.7. LC-MS Analysis, Annotation, and Identification of Top-Ranked Metabolites

Ethanol extracts were analyzed by liquid chromatography coupled to mass spectrometry using a Shimadzu LC-MS 2020 system (Shimadzu Corp., Kyoto, Japan). For this, a solution of each extract was prepared at a concentration of 5 mg/mL in absolute ethanol, filtered on a 0.22 µm pore silicone/PTFE membrane (Restek Corp., Bellefonte, PA, USA). Separation of the components of the extracts was performed on a Synergi Hydro-RP C-18 (4.6 × 150 mm^2^ and 5 μm) (Phenomenex Inc., Torrance, CA, USA) using an LC-MS system consisting of a separation module equipped with a photodiode array detector (DAD), electrospray ionization (ESI) and a detector with a quadrupole mass analyzer (Shimadzu Corp., Kyoto, Japan). The flow rate was 0.7 mL/min, and for the mobile phases, 1% formic acid in water Mili-Q and 1% formic acid in acetonitrile (ACN) were used. We prepared 1.0 µg/mL in absolute ethanol, and 10 µL of this solution was injected into the LC system. The analysis was monitored at a wavelength of 270 nm. The values of mass/charge ratio (*m/z*) were obtained under ionization in negative mode. The spectrometer parameters were configured as follows: ion spray voltage −0.5 kV; block temperature 400 ° C; drying gas flow 15 L/min (N_2_). The LC-MS-derived raw data were pre-processed with Mzmine 2.2 (Whitehead Institute for Biomedical Research, Cambridge, MA, USA) to perform the typical data pre-treatment comprising feature detection, deconvolution, filtering, deisotopization, gap-filling, gap-filled, alignment, and normalization to get list of individualized features and their peak areas [68]. Feature annotations were initially performed after detailed scrutiny of the MS data combined with ultraviolet-visible (UV-Vis) spectra of VIP-selected most contrasting metabolites (**1**–**28**, Table A2), in comparison to the chemical characteristics previously reported to *Physalis* species [16,32,33] and the information registered in the dictionary of natural products and the metlin database [69,70]. The highly sPLS-DA top-ranked compounds (**2, 6**–**9, 14,** and **23**) were finally identified using authentic standards. Quercetin (**2**) was purchased from Sigma-Aldrich (St. Louis, MO, USA). Other compounds were obtained after purification from gathered extracts by semi-preparative HPLC, using a Prominence system (Shimadzu, Columbia, MD, USA) and a reversed-phase Phenomenex Luna C18 column (250 × 10 mm^2^ and 5 μm) (Phenomenex, Torrance, CA, USA). Ten consecutive injections of target extract (400 μL, 50 mg/mL in EtOH) were separated at a flow rate of 3 mL/min using different mixtures of solvents A (1% formic acid in ACN) and B (1% aqueous formic acid) under isocratic elution. Previously annotated target peaks were collected in highly depurated fractions to afford pure compounds. Structures of purified compounds were elucidated by ^1^H and ^13^C NMR on an Agilent DD2 600 MHz spectrometer (Bruker, Billerica, MA, USA) using CDCl_3_ as solvent. NMR data of compounds **6**–**9**, **14**, and **23** coincided completely with the data of reported compounds, such as quercetin 3-O-β-glucosyl(l→6)-β-galactoside (**6**) ([71], quercetin 3-*O*-*β*-robinobioside-7-*O*-*β*-glucoside (**9**) [72], withaphysanolide (**7**) [73], physanolide A (**8**) [74], physalin B (**14**) [74], and physagulin D (**23**)[75].

### 4.8. Statistical Analysis

Normal distribution of growth parameter data was assessed by means of a Shapiro-Wilks test (*p* > 0.5). Once the normal distribution was verified, an one-way analysis of variance (ANOVA) was performed, followed by multiple comparisons through a post hoc Tukey’s HSD test to define the significant differences between treatment means, using R project software version 3.0.2 (R Foundation, Vienna, Austria). In the case of comparative analysis of metabolic profiles, the resulting whole data matrix was imported into the Metaboanalyst 4.0 (McGill University, Montreal, QC, Canada) [76]. A classical partial least squares regression with discriminant analysis (PLS-DA) was initially carried out to filter the raw dataset according to the resulting VIP scores for selecting the most contrasting features between salt treatments per growth stage. Each compared group had 12 independent replicates. This was combined with intuitive visualization through heat map distributions. Subsequently, sparse partial least squares regression with discriminant analysis (sPLS-DA) was also employed for dimension reduction, classification, and identification of spectral features that drive group separation, particularly by the selection of the top-ranked metabolites that influenced the most the specific discrimination between salt treatments, using mean centering and default parameters (5 components, 10 variable per component, and 5-fold cross-validation).

## 5. Conclusions

Studies of physiological/biochemical stress-related responses on *Physalis* plants are currently required, mainly in commercial crops such as cape gooseberry, to facilitate the identification and recognition of ecotypes and the discovery and tracking of valuable characteristics through biomarkers. In this study, the effects of salt stress on growth and metabolic profiles of the cape gooseberry were unveiled. The first agricultural implication to be used in further applications is related to the growth stage-depending plant sensitivity to salt conditions, since cape gooseberry Colombian ecotype was mostly affected by salt stress in substages 603 and 703. Additionally, we also recommend wood ash as an important pre-germination treatment since it favors a rapid and homogeneous germination of Colombian ecotype seeds under laboratory conditions. On the other hand, treated and non-treated plants displayed specific compounds that permitted statistical differentiation between treatments and control. Colombian ecotype can accumulate particular quercetin-like flavonols and non-phenolic compounds such as withanolides, depending on the growth stage and salt condition. Hence, the feature classification driving group separation led to infer that the biosynthetically-related, conjugated flavonols (differentiated by glycosylation pattern) are upregulated under mild salt stress at 209 substage, some withanolides are down-regulated at 603 and 703 substages, whereas the HS treatment promoted up-regulation of a free flavonol at all selected substages. Results suggested that the *P. peruviana* can tolerate moderate salt conditions (30 mM NaCl), and its response to salt stress (induced by 90 mM NaCl) is mediated by upregulated metabolites with antioxidant properties. However, the capacity of upregulated compounds as antioxidants, osmoregulators, and/or osmoprotectants should be studied in further experiments to delineate/explain deeper the observed responses. Our findings constitute pertinent information to be used in further studies on plant selection and breeding in order to improve the yield and characteristics of *P. peruviana* fruits as the target product.

## Figures and Tables

**Table 1:** Growth parameters of *P. peruviana* plants along three growth substages under salinized conditions.

Substage ^a^	Condition ^b^	Aerial Part		LA ^c^ (cm^2^)	Roots		TB ^c^ (g/g)
		L ^c^ (cm)	B ^c^ (g)		L ^c^ (cm)	B ^c^ (g)	
209	C	17.7 ± 1.0 ^E^	2.4 ± 0.7 ^C^	340 ± 98 ^C^	64.5 ± 3.5 ^BC^	1.2 ± 0.4 ^F^	3.7 ± 1.1 ^D^
209	LS	20.2 ± 1.0 ^E^	4.2 ± 1.2 ^C^	515 ± 149 ^C^	74.6 ± 2.2 ^A^	1.3 ± 0.4 ^F^	5.6 ± 1.6 ^D^
209	HS	20.5 ± 1.3 ^E^	2.4 ± 0.7 ^C^	371 ± 107 ^C^	70.0 ± 3.7 ^AB^	1.1 ± 0.3 ^F^	3.5 ± 1.0 ^D^
603	C	57.4 ± 1.5 ^C^	17.3 ± 5.0 ^BC^	1934 ± 558 ^B^	73.6 ± 3.3 ^A^	9.5 ± 2.8 ^C^	26.8 ± 7.7 ^CD^
603	LS	54.4 ± 2.6 ^C^	17.2 ± 5.0 ^BC^	1908 ± 551 ^B^	72.3 ± 2.5 ^A^	7.6 ± 2.2 ^CD^	24.8 ± 7.2 ^CD^
603	HS	45.2 ± 2.1 ^D^	10.9 ± 3.1 ^C^	1018 ± 352 ^C^	62.8 ± 2.8 ^BC^	4.6 ± 1.3 ^D^	15.5 ± 4.5 ^D^
703	C	92.3 ± 3.5 ^A^	53.2 ± 15.4 ^A^	3617 ± 1044 ^AB^	59.6 ± 1.8 ^C^	42.1 ± 9.1 ^A^	95.3 ± 27.5 ^A^
703	LS	81.6 ± 3.2 ^B^	45.0 ± 13.0 ^A^	3784 ± 1092 ^AB^	61.1 ± 1.3 ^C^	26.0 ± 5.5 ^B^	71.0 ± 20.5 ^AB^
703	HS	76.7 ± 3.4 ^B^	39.2 ± 11.3 ^AB^	4110 ± 1187 ^A^	60.0 ± 1.5 ^C^	15.2 ± 4.2 ^C^	54.3 ± 15.7 ^BC^

^a^ BBCH-based growth substages of *P. peruviana* [31]: vegetative (209), flowering (603), and fruiting (703); ^b^ treatment conditions: C: without NaCl treatment (control group); LS: low salinity (30 mM NaCl); HS: High salinity (90 mM NaCl); ^c^ Growth parameters: L: maximum length; B: Dry-weight biomass); LA: Leaf area; TB: Total biomass as the sum of the aerial part and roots biomasses. Data are expressed as mean ± standard error of the mean (SEM) (*n* = 12). Means with the same letter along the same column are not significantly different from each other at *p* < 0.05 according to the Tukey test.

**Table 2:** Growth parameters-derived indices for *P. peruviana* plants along growth substages under salinized conditions.

Substage ^a^	Condition ^b^	R/A ^c^ (g/g)	LA/TB ^c^ (m^2^/kg)	AMF ^c^ (g/g)	RMF ^c^ (g/g)
209	C	0.513 ± 0.145	9.3 ± 2.8	0.66 ± 0.20	0.34 ± 0.10
209	LS	0.317 ± 0.039	9.2 ± 1.7	0.76 ± 0.26	0.24 ± 0.04
209	HS	0.462 ± 0.126	10.6 ± 3.5	0.68 ± 0.24	0.32 ± 0.10
603	C	0.551 ± 0.174	7.2 ± 2.1	0.64 ± 0.19	0.36 ± 0.12
603	LS	0.438 ± 0.098	7.7 ± 2.4	0.70 ± 0.23	0.31 ± 0.08
603	HS	0.425 ± 0.142	7.9 ± 2.3	0.70 ± 0.21	0.30 ± 0.10
703	C	0.790 ± 0.449	3.8 ± 1.0	0.56 ± 0.18	0.44 ± 0.28
703	LS	0.579 ± 0.256	5.3 ± 1.4	0.63 ± 0.20	0.37 ± 0.18
703	HS	0.388 ± 0.178	7.6 ± 3.4	0.72 ± 0.22	0.28 ± 0.13

^a^ BBCH-based growth substages of *P. peruviana* [31]: vegetative (209), flowering (603), and fruiting (703); ^b^ treatment condition: C: withouht NaCl treatment (control group); LS: low salinity (30 mM NaCl); HS: High salinity (90 mM NaCl); ^c^ Growth parameters-derived indices: R/A: root/aerial part biomass ratio; LA/TB: Leaf area/total biomass ratio; AMF: aerial part mass fraction; RMF: root mass fraction. Data are expressed as mean ± standard error of the mean (SEM) (*n* = 12).

## Data Availability

The data that support the findings of this study are available from the corresponding author upon request.

## Sample Availability

Samples of the compounds are available from the authors.

## Appendix A

**Table A1:** Hoagland’s solution used for the fertigation of cape gooseberry plants throughout the growing cycle.

Nutrient	mg/L
Ca(NO_3_)_2_	861.14
KNO_3_	418.85
Agrofeed (MgNO_3_)	0.142 *
MgSO_4_	382.88
NH_4_H_2_PO_4_	119.23
Quelafeed Fe	0.03 *
Mf Mn **	4.17
Mf Cu **	0.33
Kelatex Zn **	0.78
Quibor **	1.67
(NH_4_)_6_Mo_7_O_24_	0.089

* substances measured in mL. ** Water-soluble granular fertilizers.

**Table A2:** Annotation of most contrasting metabolites based on VIP > 1 after PLS-DA analysis.

#	Rt (min)	[M − H]^−^ (*m/z*)	Annotation *^a^*	#	Rt (min)	[M − H]^−^ (m/z)	Annotation *^a^*
**1**	2.8	245	hispidin	**15**	27.0	533	withangulatin isomer
**2**	3.4	301	quercetin *^b^*	**16**	27.4	503	dihydroixocarpalactone isomer
**3**	12.8	367	feruloylquinic acid	**17**	30.5	721	physagulin isomer 1
**4**	13.4	609	quercetin rhamnosyl-glucoside	**18**	31.6	559	unidentified withanolide 1
**5**	17.6	755	kaempferol rhamnosyl-diglucoside	**19**	32.3	555	unidentified withanolide 2
**6**	18.6	771	quercetin 3-*O*-β-robinobioside-7-*O*-β-glucoside *^b^*	**20**	33.0	411	alkesterol isomer
**7**	20.1	485	withaphysanolide *^b^*	**21**	33.3	815	unknown
**8**	20.7	499	physanolide A *^b^*	**22**	34.4	515	unidentified withanolide 3
**9**	21.3	593	quercetin 3-*O*-β-glucosyl(l→6)-β-galactoside *^b^*	**23**	35.3	619	physagulin D *^b^*
**10**	23.2	547	physalin isomer	**24**	37.1	981	glycosylated triterpene
**11**	23.4	625	quercetin diglucoside	**25**	37.6	705	physagulin isomer 2
**12**	24.1	521	deoxyphysalolactone isomer	**26**	39.2	809	unknown
**13**	24.4	473	pubescenol	**27**	39.8	735	unidentified withanolide 4
**14**	26.8	509	physalin B *^b^*	**28**	41.4	719	unidentified withanolide 5

*^a^* Annotation was accomplished based on the MS data combined with UV-Vis spectra; *^b^* Statistically-selected metabolites identified using authentic standards as described in this manuscript (Materials and Methods, Section 4.7).
